# Distinct Transcriptomic Features are Associated with Transitional and Mature B-Cell Populations in the Mouse Spleen

**DOI:** 10.3389/fimmu.2015.00030

**Published:** 2015-02-11

**Authors:** Eden Kleiman, Daria Salyakina, Magali De Heusch, Kristen L. Hoek, Joan M. Llanes, Iris Castro, Jacqueline A. Wright, Emily S. Clark, Derek M. Dykxhoorn, Enrico Capobianco, Akiko Takeda, Ryan M. McCormack, Eckhard R. Podack, Jean-Christophe Renauld, Wasif N. Khan

**Affiliations:** ^1^Department of Microbiology and Immunology, Miller School of Medicine, University of Miami, Miami, FL, USA; ^2^Center for Computational Science, University of Miami, Miami, FL, USA; ^3^Ludwig Institute for Cancer Research, Brussels Branch, Brussels, Belgium; ^4^de Duve Institute, Université Catholique de Louvain, Brussels, Belgium; ^5^Department of Pathology, Microbiology and Immunology, Vanderbilt University School of Medicine, Nashville, TN, USA; ^6^Hussman Institute for Human Genomics, University of Miami, Miami, FL, USA; ^7^Department of Pathology and Immunology, Washington University School of Medicine in St. Louis, St. Louis, MO, USA

**Keywords:** transcriptome by RNA-seq technique, splenic transitional B-cells, follicular 1 and 2 B-cells, marginal zone B-cells, DAP10 PI3K pathway, IL-9/IL-9R, Myb Myc, Toll-like receptors 3 and 7

## Abstract

Splenic transitional B-cells (T1 and T2) are selected to avoid self-reactivity and to safeguard against autoimmunity, then differentiate into mature follicular (FO-I and FO-II) and marginal zone (MZ) subsets. Transcriptomic analysis by RNA-seq of the five B-cell subsets revealed T1 cell signature genes included RAG suggesting a potential for receptor revision. T1 to T2 B-cell differentiation was marked by a switch from Myb to Myc, increased expression of the PI3K adapter DAP10 and MHC class II. FO-II may be an intermediate in FO-I differentiation and may also become MZ B-cells as suggested by principle component analysis. MZ B-cells possessed the most distinct transcriptome including down-regulation of CD45 phosphatase-associated protein (CD45-AP/PTPRC-AP), as well as upregulation of IL-9R and innate molecules TLR3, TLR7, and bactericidal Perforin-2 (MPEG1). Among the endosomal TLRs, stimulation via TLR3 further enhanced Perforin-2 expression exclusively in MZ B-cells. Using gene-deleted and overexpressing transgenic mice we show that IL-9/IL-9R interaction resulted in rapid activation of STAT1, 3, and 5, primarily in MZ B-cells. Importantly, CD45-AP mutant mice had reduced transitional and increased mature MZ and FO B-cells, suggesting that it prevents premature entry of transitional B-cells to the mature B-cell pool or their survival and proliferation. Together, these findings suggest, developmental plasticity among splenic B-cell subsets, potential for receptor revision in peripheral tolerance whereas enhanced metabolism coincides with T2 to mature B-cell differentiation. Further, unique core transcriptional signatures in MZ B-cells may control their innate features.

## Introduction

The role of B-lymphocytes is to produce antigen specific antibodies to neutralize pathogens. B-cells develop in the bone marrow (BM) where most autoreactive clones are triaged by the central tolerance mechanisms of clonal deletion, anergy, or receptor editing (1). Surviving sIgM^+^ immature or transitional 1 (T1) B-cells migrate to the spleen, where they are again tested for autoreactivity. Innocuous clones are allowed to develop into transitional 2 (T2) cells (2). Observations that T1 cells are extremely sensitive to BCR-induced apoptosis *in vitro* suggest that the T1-stage serves as a peripheral tolerance checkpoint (3–7). Dysregulation of peripheral checkpoint can lead to autoimmune pathologies such as SLE, RA, and MS (8–10).

The immature T2 cell stage is believed to serve as the branching point for selection into functionally distinct mature B-cell subsets comprised of follicular I and II (FO-I and FO-II), B1, and marginal zone (MZ) B-cell compartments [reviewed in Ref. (11)]. FO-I cells specialize in T cell-dependent (TD) immune responses whereas MZ B-cells specialize in rapid T cell-independent (TI) antibody responses and possess innate-like properties (11–13). The function of the FO-II subset is unknown (14). A comprehensive analysis to identify transcriptional changes associated with peripheral tolerance at the transitional stages and functional specialization of mature B-cell subsets may provide a framework for hypothesis-driven experiments to identify key processes responsible for B-cell biological properties.

The Immunological Genome consortia (ImmGen) has provided a rich resource for gene expression data sets to the immunological community including all known mouse B-cell subsets using microarray. Analyses of these gene expression data sets have produced gene-network models laying the foundation for experimentally testable hypotheses for various hematopoietic lineage cell developmental relationships and acquisition of functional specialization. However, such analysis has not been reported for the B lineage. Here, we report bioinformatics analysis performed on data obtained with next generation sequencing (NGS) on highly purified B-cell subsets that are either not available from ImmGen (FoB-II) or were phenotypically defined differently than the current study. Our splenic B-cell populations were enriched using a combination of schemes and to achieve maximum cell homogeneity defined as; T1^21/23DN^ (B220^+^, AA4.1^+^, CD23^−^, CD21^−^, CD24^hi^), T2^CD21int^ (B220^+^, AA4.1^+^, CD23^+^, CD21^int^, CD24^hi^), FO-I (B220^+^, IgM^lo^, CD21^int^, IgD^+^, CD23^+^, CD24^lo^, CD9^−^), FO-II (B220^+^, IgM^hi^, CD21^int^, IgD^+^, CD23^+^, CD24^lo^, CD9^−^), and MZ^CD9+^ (B220^+^, IgM^hi^, CD21^hi^, IgD^−^, CD23^−^, CD24^int^, CD9^+^).

We identified many novel stage-specific transcripts not identified by ImmGen data sets and associated processes. Our comparative analysis of transcriptomes in specific B-cell subsets has advanced our understanding of the transcriptional networks associated with peripheral B-cell development and selection as well as functional specialization acquired by mature B-cell subsets. We highlight transcripts contributing to innate MZ B-cell function (TLR3 and Perforin-2) and demonstrate a previously unknown function for IL-9R and CD45-AP in B-cells.

## Materials and Methods

### Mice

C57BL/6 mice were purchased from The Jackson Laboratory and maintained at University of Miami animal facility. *CD45-AP*^−^*^/^*^−^(C57BL/6), IL-9R^−/−^, and IL-9 transgenic (Tg5) mice have been previously described (15–17). Unless indicated otherwise, mice used in these studies were aged between 6 and 10 weeks. These studies were approved by the Institutional Animal Care and Use Committee.

### Flow cytometric analysis and cell sorting

CD93^+^ enriched immature and CD93^+^CD43^+^ depleted mature splenocytes were incubated with fluorochrome labeled antibodies to sort T1^21/23DN^, T2^CD21int^, FO-I, FO-II, and MZ^CD9+^ on a BD FACS Aria II yielding 95–99% purity as described in Figure S1 in Supplementary Material. Briefly, B-cell enrichment was performed using Mouse B Lymphocyte Enrichment set-DM (BD Biosciences). Transitional B-cells were sorted from pooled splenocytes after two rounds of AA4.1 positive selection. Sorting from DAPI^−^, B220^+^ cells produced two relatively homogeneous transitional subsets. T1^21/23DN^ were AA4.1^+^, CD23^−^, CD21^−^, CD24^hi^, whereas T2^CD21int^ were AA4.1^+^, CD23^+^, CD21^int^, CD24^hi^. For mature B-cell sorting, pooled splenocytes were enriched for B-cells and simultaneously depleted of AA4.1^+^ (transitional) cells. Three purified mature B-cell populations were sorted from the DAPI^−^, B220^+^ gate. FO-I were IgM^lo^, CD21^int^, IgD^+^, CD23^+^, CD24^lo^, and CD9^−^. FO-II were IgM^hi^, CD21^int^, IgD^+^, CD23^+^, CD24^lo^, and CD9^−^. MZ^CD9+^ were IgM^hi^, CD21^hi^, IgD^−^, CD23^−^, CD24^int^, and CD9^+^.

Flow cytometric analysis of splenocytes from CD45-AP-deficient mice and IL-9R^−/−^/IL-9 transgenic were performed on a LSRII Flow cytometer and LSR Fortessa, respectively (BD Bioscience). For intracellular staining, cells were stimulated 15 min with IL-9 for 15 min. Cells were then fixed 10 min in 2% PFA, 90% methanol permeabilized (30 min), washed extensively, blocked and then incubated 1 h with anti-pSTAT, and surface marker antibodies. FCM data were analyzed using FlowJo software (TreeStar). Antibodies used are listed as follows; CD93 Biotin Clone AA4.1, CD23 PE/Biotin Clone B3B4, CD21 FITC/PE Clone 7G6, Streptavidin PE Cy7, B220 V500 Clone RA3-6B2, CD19 Clone 1D3, CD9 Biotin Clone KCM8, pSTAT3 (pY705) Alexa Fluor 647 Clone 4/pSTAT3, pSTAT1 (pY701) Alexa Fluor 647 Clone 4a, pSTAT5 (pY694) Alexa Fluor 647 Clone 47/Stat5 (pY694) (BD Bioscience), CD93 APC Clone AA4.1, CD24 PerCP Cy5.5 Clone M1/69, IgD PacBlue Clone 11-26 (eBioscience), B220 Alexa 700 Clone RA3-6B2, Streptavidin PerCP (Biolegend), IgM 649 Fab Fragment (Jackson ImmunoResearch), CD9 FITC Clone MZ3 (Santa Cruz Biotechnology), DAPI (Invitrogen). B220 Alexa 700, CD9 Biotin, Streptavidin PerCP, and pSTAT antibodies were used for IL-9R/IL-9 studies. CD21 Clone 7G6 is used for both sorting and IL-9R/IL-9 studies.

### RNA isolation for next generation sequencing and analysis

RNA was prepared from the sorted cells using Qiagen RLT buffer. PolyA RNA was selected and library constructed using Illumina RNA sample preparation reagents following manufacturer’s recommendations (Illumina). RNA integrity was assessed using a Bioanalyzer 2100 (Agilent) as well as Nanodrop 8000 Spectrophotometer (Thermo Scientific). Four of the five samples were sequenced on the Illumina GAIIx using Cluster Generation Kit v4 and Sequencing Kit v4, generating 74base single-end reads. The fifth sample (MZ^CD9+^) was sequenced on the Illumina HiSeq2000 using the reagents provided in the Illumina TruSeq PE Cluster Kit v3 and the TruSeq SBS Kit – HS (200 cycle) kit, generating 99base paired-end reads. Read2 was not used in this study and read1 was trimmed to match the 74base reads generated by the GAIIx. Quality of the RNA-seq data was reviewed using FastQC software version 0.10.1^1^. Average phred-like quality scores were >30 in all samples if calculated per-base and over 36 if calculated per sequence. This quality was considered sufficient and no reads were filtered out. All samples passed testing on basic parameters in FastQC (data not shown), except sequence duplication levels (Table S1 in Supplementary Material). Elevated duplication levels may arise in RNA-seq due to “over-sequencing” of high abundant transcripts as well as bias caused by non-random hexamer priming (18). We did not remove any duplicates because there is no consensus so far how it affects expression level estimations. Table S1 in Supplementary Material shows basic statistics on the samples.

Quantification of transcriptome was done in two steps as described in TopHat protocol (19). In the first step, TopHat was used to map the reads to the reference genome (UCSC build mm10 GRCm38 from September 2012) with default settings and novel splice discovery disabled. In the second step, Cufdiff was employed to calculate FPKM values using reference transcriptome along with BAM files from the first step for each sample. Data were analyzed in automated fashion on the cluster hosted by the High-Performance Computing core in the Center for Computational Science, University of Miami. All transcript expression below FPKM 1 was set to 1. Quantitative FPKM values were log2 transformed and converted to Z-scores. miRNA, rRNA, and hemoglobin transcripts were removed from the analysis as these transcripts likely represented artifacts (20). Table S1 in Supplementary Material contains FPKM, log2 transformed FPKM, *z*-scores, and fold-change (FC) relative to T2^CD21int^.

RNA species compositions (biotypes) were analyzed and visualized using NOIseq package in Bioconductor^2^. Biotypes from Ensemble annotation were used (Figure S1 in Supplementary Material). Data analysis was performed using R software^3^. Scatterplot was generated using “pairs” R function. Principle component analysis (PCA) was performed with “prcomp” R function. *Z*-scores for gene expression were visualized as heatmaps using “heatmap.2” function from “gplots” R library. Venn diagrams were generated using “VennDiagram” library in R. Functional annotation clustering was done using DAVID bioinformatics online software (21, 22). Each term is ranked based on enrichment score along with corresponding *P* value. Prioritization of clusters was based on enrichment score using highest stringency settings. GeneGo software (MetaCore, Thomson Reuters) was used to predict transcription factor (TF) regulation during development. All differentially expressed (DE) genes (FC > 2) between two subsets (or signature genes) were used as input.

### Real-time PCR

RNA for quantitative Real-Time PCR (qRT-PCR) was isolated using RNeasy Minikit and reverse-transcribed utilizing Quantitect Reverse Transcription kit (Qiagen). qRT-PCR was performed with TaqMan Fast Universal PCR Master Mix in Step One Real-Time PCR System (Applied Biosystems). TaqMan primer/probes (Applied Biosystems) are as follow: Gfi1 Mm00515855_m1, Tlr3 Mm01207404_m1, Tlr7 Mm00446590_ m1, Tlr9 Mm00446193_m1, Rag1 Mm01270936_m1, Rag2 Mm01270938_m1, IL-9R Mm0043413_m1, Tnfrsf13c/BAFF-R Mm00840578_g1, Ptprc-ap/CD45-AP Mm01236556_m1, Mpeg1/Perforin-2 Mm01222137_g1, Hcst/Dap10 Mm01270936_m1, Bmf Mm00506773_m1, IKKε Mm00444862_m1, Tnfrsf13b/TACI Mm00840182_m1, and Gapdh Mm99999915_g1.

### Western blot

T1, T2, and Mature B-cell (FO) subsets were sorted and western-blotted as previously described in Ref. (7). Briefly, 20 μg/lane of total cellular extracts from FACS cells were analyzed by immunoblotting with antibodies specific for the indicated anti-apoptotic proteins. Anti-p38 was used as a loading control. Antibody information is as follows: Mcl-1 (Rockland Immunochemicals); Bcl-2, Bcl-xL, p38, Pim-2 (Santa Cruz Biotechnology); A1 (R&D Systems); and c-IAP2 (Cell Signaling Technology).

### B-cell isolation for *in vitro* stimulation

Splenic B-cells enriched by CD43 negative selection or CD45/B220 positive selection beads (BD Bioscience) were cultured (7) and stimulated with Poly(I:C) (#tlrl-pic), CL097 (#tlrl-c97), or CpG (tlrl-1826) from Invivogen.

### Statistical analysis

Biological data were analyzed using Student’s *t*-test. All data are represented as mean ± SEM. Values of *P ≤ 0.05 were considered statistically significant. Analysis performed using Prism software (GraphPad).

## Results

### Transcriptome analysis reveals previously unknown relationships among splenic B-cell subsets

**Figure 1:**
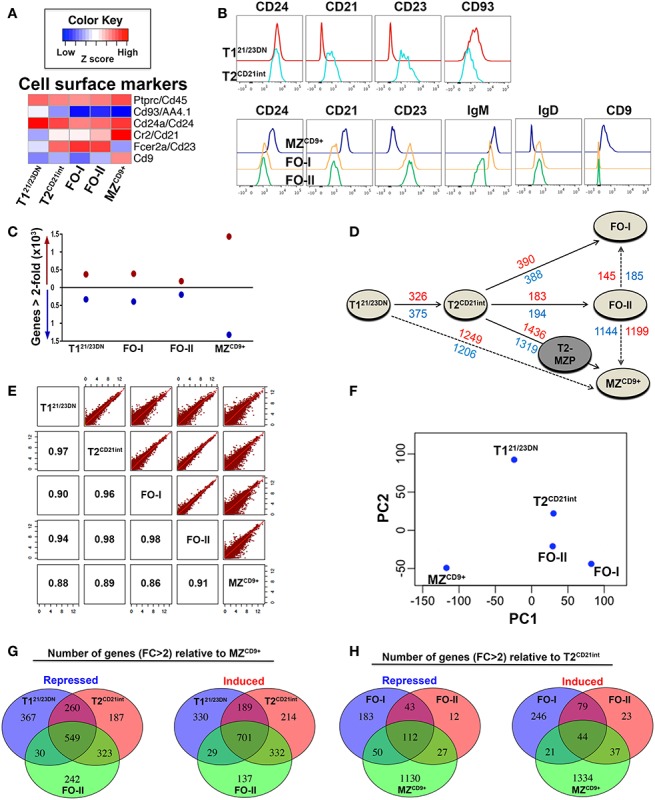
**Global gene expression profiles define hierarchical relationships among splenic B-cell subsets**. **(A)** NGS results of gene expression profiles of each B-cell subset corroborate well with the cell-surface marker expression shown in **(B)**. Heatmap displaying expression of mRNAs (median log-transformed relative fold-change) encoding cell-surface markers that define T1^21/23DN^, T2^CD21int^, FO-I, FO-II, and MZ^CD9+^ B-cell subsets and were used for cell sorting by FACS (see Figure S1 in Supplementary Material for details). Immunoglubulin sequences were excluded from NGS data analysis. Red color indicates high *z*-score (high expression) while blue indicates a low *z*-score (low expression). **(B)** Histograms depict cell-surface marker expression that define transitional (top panels) and mature (bottom panels) splenic B-cell subsets as in **(A)**. **(C)** Graphic depiction of the number of genes differentially expressed (DE) relative to T2^CD21int^ (FC > 2). Red circles (above horizontal line at 0) represent the number of up-regulated genes while the blue circles (below horizontal line at 0) represent the number of down-regulated genes. T1^21/23DN^, FO-I, FO-II, and MZ^CD9+^ had 6.46, 7.17, 3.74, and 25.39% DE genes (includes up- and down-regulated), respectively. **(D)**, Distance plot indicating the number of up-regulated genes (red) and down-regulated genes (blue) in various maturation steps (FC > 2). Solid arrows indicate known paths of maturation whereas dotted arrows indicate alternate maturation paths predicted by NGS data analysis in this study. Although we did not sort T2-MZP (gray), it is included for completeness. It is believed to be an intermediate between T2 to MZ B-cell differentiation. **(E)** Scatterplot analysis of log-transformed NGS data showing similarity in global gene expression between all B-cell subsets. Numbers inside intersecting boxes indicate the percentage of similarity. Graphical representation of this percentage is displayed on upper panels where each red dot corresponds to a single gene. **(F)** Principle component analysis (PCA) using two principle components (PC1 and PC2) where PC1 explained the majority of gene expression differences followed by PC2. Similarity between B-cell subsets can be ascertained by the physical difference between circles on the plot. Both scatterplot and PCA analysis do not involve fold-change cut-offs and involve all subset cross comparisons. **(G)** The number of repressed or induced genes overlapping during differentiation from either T1^21/23DN^, T2^CD21int^, or FO-II to MZ^CD9+^ cells. **(H)** The number of repressed or induced genes overlapping in differentiation from T2^CD21int^ to either FO-I, FO-II, or MZ^CD9+^ cells.

**Figure 2:**
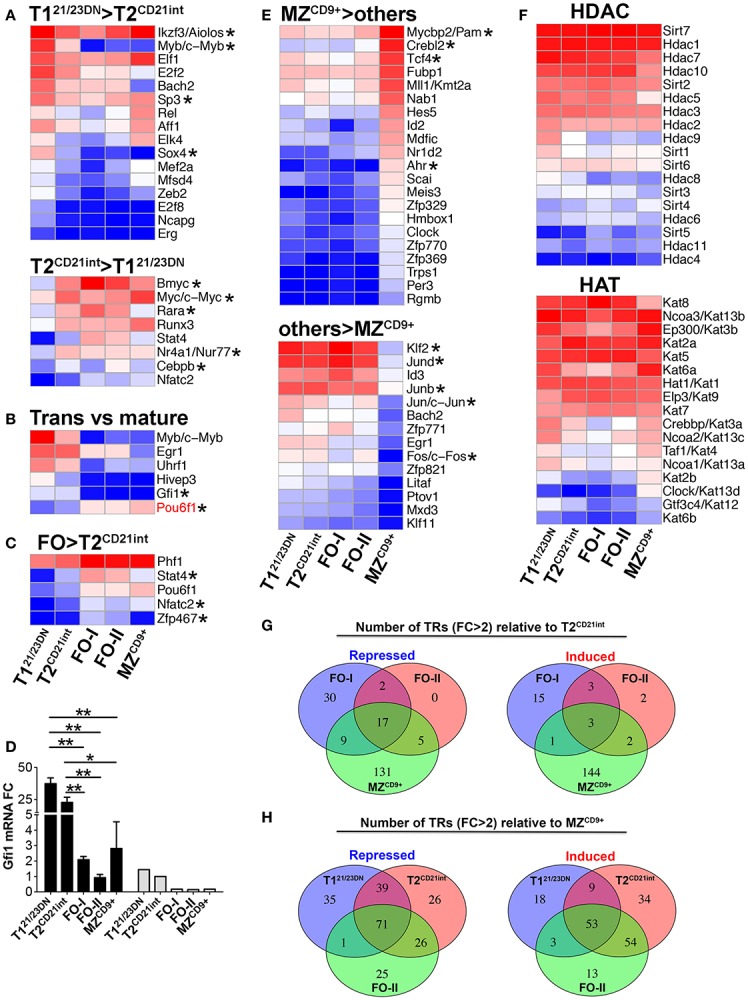
**Expression of distinct transcription regulators in splenic B-cell subsets**. Heatmap display of NGS analysis of DE transcription regulators (including TFs) using *z*-scores comparing: **(A)** T1^21/23DN^ to T2^CD21int^ (FC > 3 and select FC > 2); **(B)** transitional cells (T1^21/23DN^ and T2^CD21int^) to all mature B-cells (FC > 3); **(C)** FO (FO-I and FO-II) to T2^CD21int^ (FC > 2); and **(E)** MZ^CD9+^ to all other subsets (FC > 3), up-regulated (top heatmap) and down-regulated (bottom heatmap). Pou6f1, the only gene over threefold higher in mature relative to transitional cells, is shown in red **(B)**. Asterisks denote important genes discussed in the text. Many of the TFs shown are predicted to regulate genes that are DE in the B-cell subsets (refer to Table S3 in Supplementary Material). **(D)** Gfi1 is down-regulated during transitional to mature B-cell differentiation. qRT-PCR (black bars) validation of NGS results (gray bars). qRT-PCR data are representative of three independent experiments with FACS-sorted B-cell subsets, where each experiment utilized pooled splenocytes from 2 to 5 mice. *P ≤ 0.05; **P ≤ 0.01. **(F)** Heatmap display of transcripts encoding histone deacetylase (HDAC, top) and histone acetylase (HAT, bottom) enzymes. **(G)** The number of repressed or induced TRs that overlap during differentiation from T2^CD21int^ to either FO-I, FO-II, or MZ^CD9+^ cells. **(H)** The number of repressed or induced genes that overlap during differentiation from either T1^21/23DN^, T2^CD21int^, or FO-II to MZ^CD9+^ cells.

**Figure 3:**
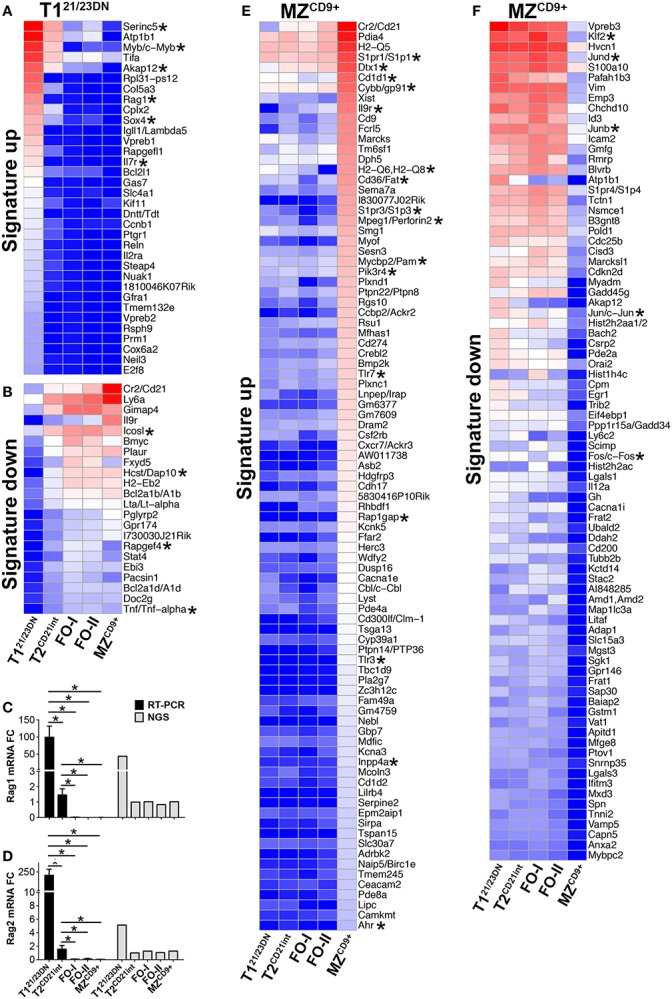
**T1^21/23DN^ and MZ^CD9+^ B-cell subsets are highly enriched for signature genes**. **(A–D)** RAG1 and RAG2 transcripts are prominent T1^21/23DN^ signature genes. Heatmap displaying *z*-scores of T1^21/23DN^ signature genes (FC > 4) that are uniquely up-regulated **(A)** or down-regulated **(B)**. qRT-PCR analysis validated NGS results showing RAG1 and RAG2 are up-regulated T1^21/23DN^ signature genes. **(C)** RAG1 mRNA expression by qRT-PCR (black bars) and RNA-Seq (gray bars) and **(D)** RAG2 mRNA expression analyzed as in **(C)**. qRT-PCR data represent three independent experiments with FACS-sorted B-cell subsets, where each experiment utilized pooled splenocytes from 2 to 5 mice *p ≤ 0.05. **(E,F)** MZ^CD9+^ B-cells show highest number of signature genes. Heatmap displaying *z*-scores of MZ^CD9+^ (FC > 4) that are uniquely up-regulated **(E)** or down-regulated **(F)**. Asterisks in heatmaps denote genes discussed in the text. Due to the high number of up-regulated signature genes in MZ^CD9+^ cells, genes below Ahr (57 out of 146) were removed but can be referenced in Table S1 in Supplementary Material.

**Figure 4:**
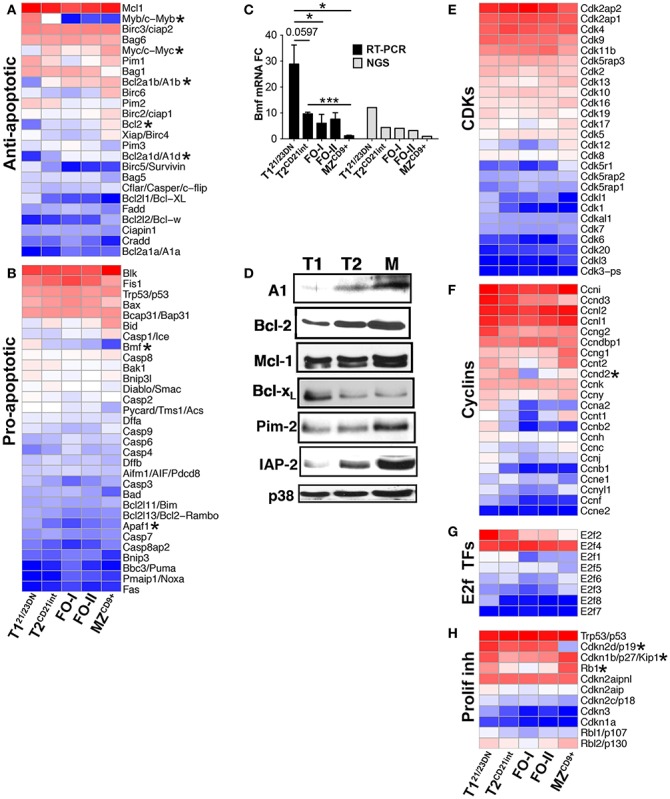
**Differentially expressed genes define apoptotic and proliferative potential of transitional and mature B-cell subsets**. Heatmap display of NGS data analysis of genes encoding **(A)** anti- apoptotic, **(B)** pro-apoptotic, **(E)** cyclin dependent kinases (CDKs), **(F)** cyclins, **(G)** E2F cell cycle transcription factors, and **(H)** proliferation inhibitors, using *z*-scored NGS gene expression data. Asterisks denote important genes discussed in the text. **(C)** qRT-PCR analysis (black bars) validated NGS results (gray bars) showing upregulation of Bmf in T1^21/23DN^ cells. qRT-PCR data are representative of three independent experiments with FACS-sorted B-cell subsets, where each experiment utilized pooled splenocytes from 2 to 5 mice. *P ≤ 0.05; **P ≤ 0.01; ***P ≤ 0.001. **(D)** Western blot of anti-apoptotic proteins showing key survival factors such as Bcl-2 increase during differentiation. p38 served as a loading control.

**Figure 5:**
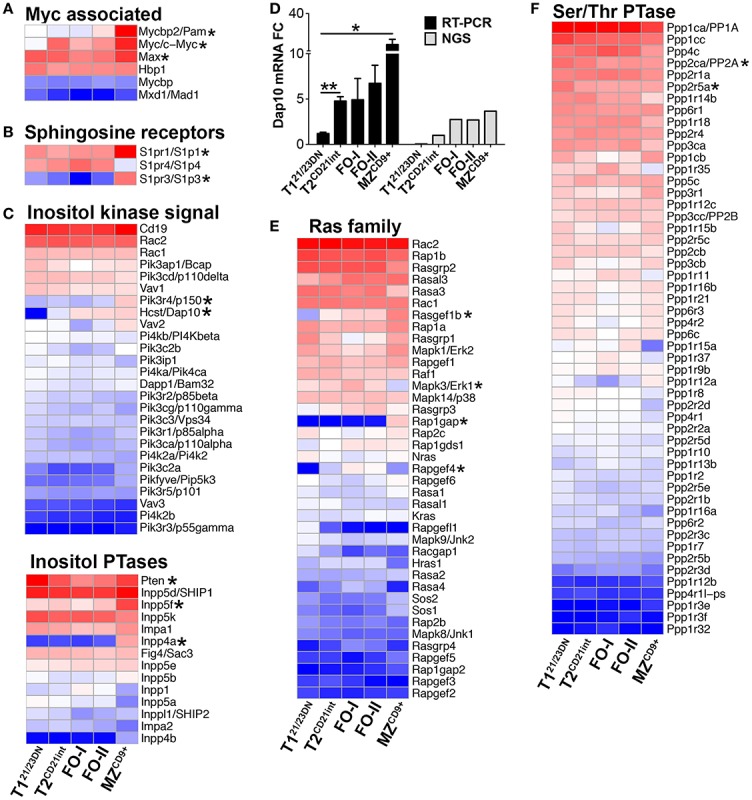
**Gene expression profiles predict PI3K/Ras signaling stabilizes Myc during B-cell development**. Heatmap display of **(A)** Myc associated genes including Myc binding partners Pam and Max, **(B)** sphingosine receptors possibly mediating Pam/mTOR signaling, **(C)** inositol kinase signaling molecules including Dap10 (bottom) as well as inositol phosphatases (bottom), **(E)** Ras family genes, and **(F)** Serine/threonine phosphatases. **(D)** qRT-PCR analysis (black bars) validated NGS results (gray bars) showing upregulation of Dap10 starting at the T2^CD21int^ stage. qRT-PCR data are representative of three independent experiments with FACS-sorted B-cell subsets, where each experiment utilized pooled splenocytes from 2 to 5 mice. *P ≤ 0.05; **P ≤ 0.01.

**Figure 6:**
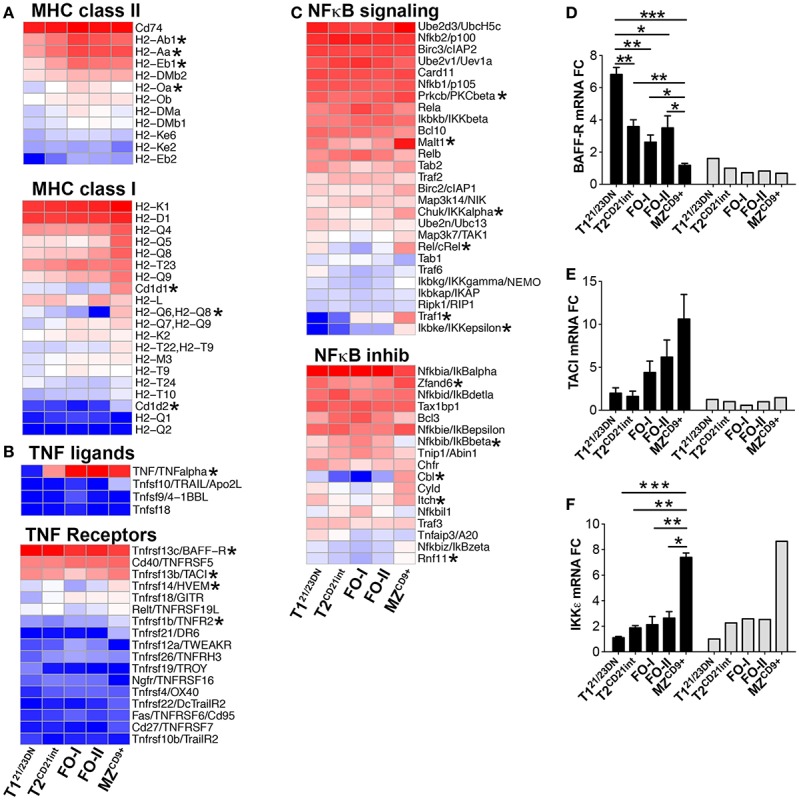
**(A,B)** Expression of genes associated with immune competence from the T2^CD21int^ stage and **(C–F)** upregulation of genes associated with activated and innate characteristics of MZ^CD9+^ cells. Heatmap display of **(A)** MHC class II (top) and MHC class I (bottom), **(B)** TNF ligands (top) and TNF receptors (bottom), and **(C)** NFκB signaling components (top) and NFκB inhibitors (bottom). qRT-PCR analysis (black bars) validated NGS results (gray bars) of **(D)** BAFF-R, **(E)** TACI, and **(F)** IKKε. qRT-PCR data are representative of three independent experiments with FACS-sorted B-cell subsets, where each experiment utilized pooled splenocytes from 2 to 5 mice. *P ≤ 0.05; **P ≤ 0.01.

**Figure 7:**
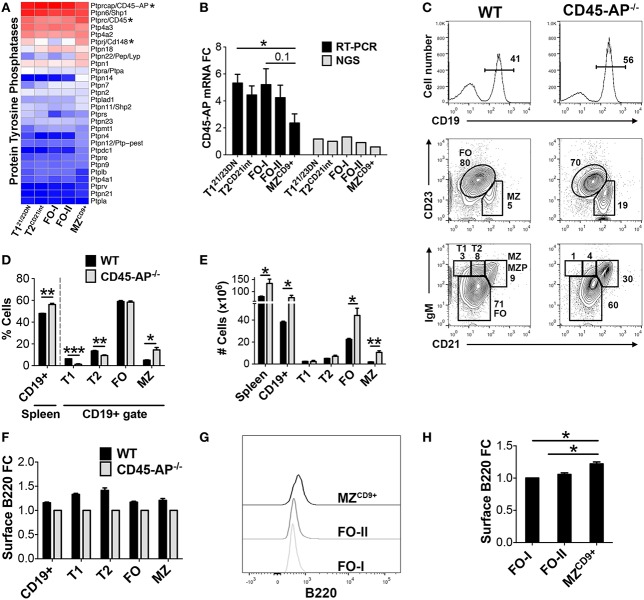
**CD45-AP limits premature entry of transitional B-cells into the mature B-cell pool**. **(A)** Heatmap display of protein tyrosine phosphatases including tyrosine phosphatase PTPN22, CD45/Ptprc, and CD45 binding partner CD45-AP/Ptprc-ap (astericks). **(B)** qRT-PCR analysis validated NGS results showing CD45-AP is down-regulated in MZ^CD9+^ B-cells. CD45-AP mRNA expression by qRT-PCR (black bars) and RNA-Seq (gray bars). qRT-PCR data are representative of three independent experiments with FACS-sorted B-cell subsets, where each experiment utilized pooled splenocytes from 2 to 5 mice. **(C)** Phenotypic analysis of splenic B-cells isolated from WT and CD45-AP^−/−^ mice. Histogram showing percent of CD19^+^ B-cells within splenocytes (top panels). CD19^+^ gated B-cells were further analyzed for transitional (T1 and T2), FO, and MZ B-cells. CD23 versus CD21 staining is used to differentiate MZ (CD21^hi^, CD23^lo^) and FO (CD21^lo-int^, CD23^+^). IgM versus CD21 allows for analysis of additional subsets such as T1 (IgM^hi^, CD21^−^), T2 (IgM^hi^, CD21^lo-int^), and preMZ (MZP). Data are representative of three mice. Bar graph representation of B-cell subset percentages **(D)** and numbers from three mice **(E)**. **(F)** Absence of CD45-AP modestly affects cell-surface display of B220 protein. FCM analysis of WT versus CD45-AP^−/−^ splenic B-cell subsets for the expression of B220. **(G)** MZ^CD9+^ have the highest surface expression of B220. Post-sort surface B220 expression of sorted FO-I, FO-II, and MZ^CD9+^ B-cells (refer to Figure S1 in Supplementary Material) and plotted in **(H)** bar graph. *P ≤ 0.05; **P ≤ 0.01; ***P ≤ 0.001.

**Figure 8:**
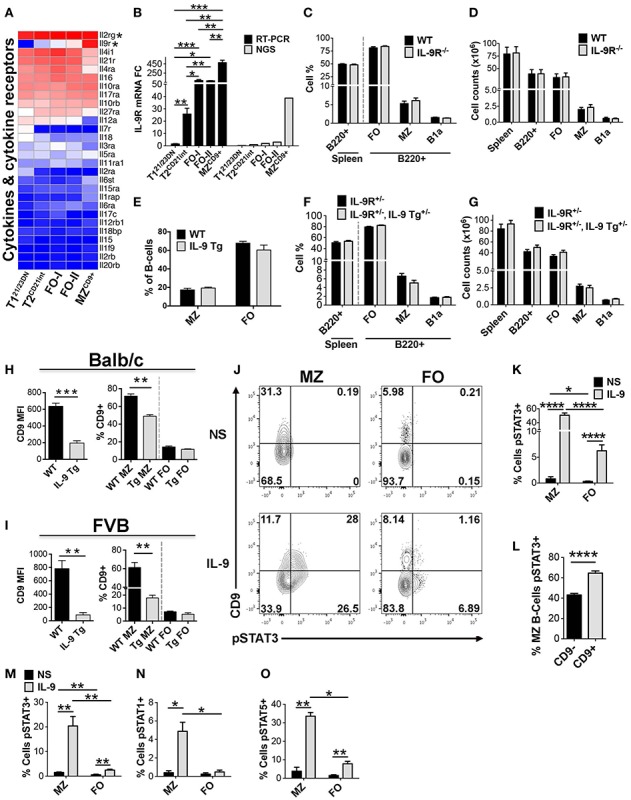
**Increased IL-9R expression manifests in enhanced responsiveness of MZ^CD9+^ B-cells to IL-9**. Increased IL-9R expression manifests in enhanced responsiveness of MZ^CD9+^ B-cells to IL-9. **(A)** Heatmap display of cytokine and cytokine receptors including IL-9R and its heterodimerization partner IL-2Rg (astericks). **(B)** qRT-PCR analysis validated NGS results showing IL-9R is a MZ^CD9+^ up-regulated signature gene. IL-9R mRNA expression by qRT-PCR (black bars) and RNA-Seq (gray bars). qRT-PCR data are representative of three independent experiments with FACS-sorted B-cell subsets, where each experiment utilized pooled splenocytes from 2 to 5 mice. **(C–D)** IL-9R is dispensable for splenic B-cell development. Bar graph displaying MZ (B220^+^, CD21^hi^, CD23^lo^, IgM^hi^) and FO (B220^+^, CD21^lo-int^, CD23^hi^, IgM^+^) B-cell subset percentages **(C)** and numbers **(D)** from Balb/c control versus IL-9R^−/−^ mice. FO represents follicular B-cell populations (FO-I, FO-II, and T2 B-cells). B1a B-cells were gated as (B220^+^, CD23^−^, IgM^hi^, CD5^+^). Data are representative of six mice. Transgenic over-expression of IL-9 does not affect B-cell development. **(E)** Bar graph displaying MZ (B220^+^, CD21^hi^, CD23^lo^, IgM^hi^) and FO (B220^+^, CD21^lo-int^, CD23^hi^, IgM^+^) B-cell percentages from FVB WT control and IL-9 transgenic (Tg5) mice. Data are representative of three mice. **(F,G)** Bar graph displaying MZ (B220^+^, CD21^hi^, CD23^lo^, IgM^hi^) and FO (B220^+^, CD21^lo-int^, CD23^hi^, IgM^+^) B-cell subset percentages **(F)** and numbers **(G)** from Balb/c IL-9R^+/−^ control mice and IL-9 transgenic (Tg5) heterozygotes (IL-9R^+/−^, IL-9 Tg^+/−^) mice. Data are representative of six mice. **(H,I)** MZ B-cells display reduced cell-surface CD9 in transgenic mice overexpressing IL-9. Left bar graphs show CD9 mean fluorescence intensity (MFI) of MZ B-cells (B220^+^, CD21^hi^, CD23^lo^) comparing WT versus IL-9 Tg mice. Right bar graphs show the percentage of cells displaying CD9^+^ positivity comparing MZ versus FO B-cells (B220^+^, CD21^lo-int^, CD23^+^). Bar graphs in H and I are representative of three mice. **(J,K)** Short term IL-9 induces STAT3 activation selectively in MZ B-cells. **(J)** FCM analysis of pSTAT3 in FVB splenic MZ (B220^+^, CD21^hi^, CD23^lo^) versus FO (B220^+^, CD21^lo-int^, CD23^+^) B-cells stimulated or non-stimulated (NS) with bacculo-produced IL-9 (3.5 ng/mL) for 15 min at 37°C. FO represents follicular B-cell populations (FO-I, FO-II, and T2 B-cells). Numbers in quadrants represent the percentage of cells. **(K)** Bar graph comparing the percentage of pSTAT3 positive cells in MZ versus FO B-cells. **(L)** Bar graph comparing the percentage of pSTAT3 positive cells in CD9^+^ versus CD9^−^ MZ B-cells. Data from **(H–J)** are representative of five mice. **(M–O)** IL-9 induction of pSTAT3 is independent of genetic background. Balb/c wild-type mice were treated as in J and stained for either pSTAT3, pSTAT1, or pSTAT5. pSTAT3 data **(M)** represent three mice whereas pSTAT1 **(N)** and pSTAT5 **(O)** data represent two mice. *P ≤ 0.05; **P ≤ 0.01; ***P ≤ 0.001; ****P ≤ 0.0001.

**Figure 9:**
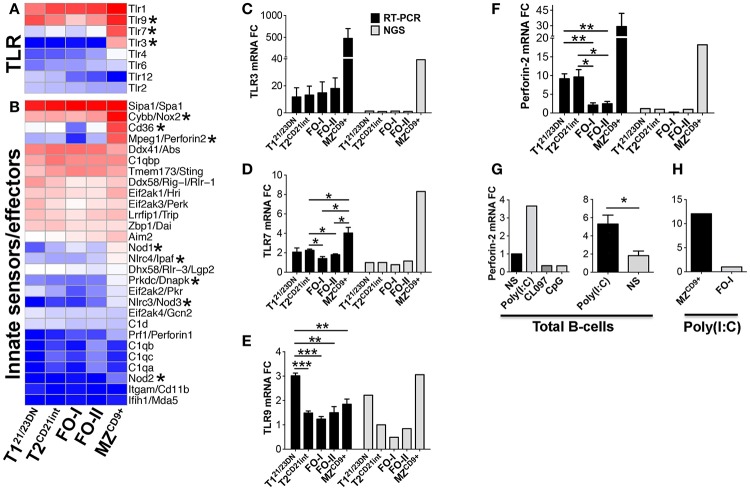
**MZ^CD9+^ B-cell subset is poised for innate immune responses**. Heatmap display of NGS data showing enrichment of **(A)** TLR and **(D)** innate immune sensors/effector genes. Asterisks denote important genes discussed in the text. qRT-PCR analysis validated NGS results showing MZ^CD9+^ express highest amounts of TLR3, TLR7 and Perforin-2. qRT-PCR (black bars) and RNA-Seq (gray bars) of **(B)** TLR3, **(C)** TLR7, and **(E)** Perforin-2. qRT-PCR data are representative of three independent experiments with FACS-sorted B-cell subsets, where each experiment utilized pooled splenocytes from 2 to 5 mice. **(F)** qRT-PCR of Perforin-2 mRNA fold-change after stimulation with endosomal TLR agonists. B220 positively selected cells (>95% purity) were stimulated 18 h with either TLR3 agonist Poly(I:C), TLR7/8 agonist CL097, or TLR9 agonist CpG at 1 μg/mL and compared to NS. **(G)** qRT-PCR of Perforin-2 mRNA fold-change using B220 positive (>95% purity) or CD43 negative (~90% purity) B-cells stimulated with Poly(I:C) as in **(F)**. Data are representative of four independent experiments. **(H)** qRT-PCR of Perforin-2 mRNA fold-change using FACS derived MZ^CD9+^ or FO-I B-cells treated with Poly(I:C) as in **(F)**. *P ≤ 0.05; **P ≤ 0.01.

We FACS-purified five splenic B-cell subsets using a combination of schemes previously described for mouse and human B-cells (2, 4, 6, 14, 23–25). This purification scheme was used to maximize homogeneity for RNA-Seq analysis (Figure S1 in Supplementary Material). Hereafter, B-cell subsets will be referred to as T1^21/23DN^, T2^CD21int^, FO-I, FO-II, and MZ^CD9+^ to denote distinction from other sorting schemes. As shown in Figures 1A,B, the NGS data corroborate well with cell-surface marker expression used in sorting thereby confirming both phenotype and purity. qPCR further verified the accuracy of our NGS data (Figures 2–9).

Transcriptome analysis yielded 10,851 unique transcripts among our subsets. Approximately 19% (2057) of transcripts were up-regulated and 16.5% (1789) were down-regulated (FC > 2) in at least one subset relative to T2^CD21int^. This subset was used for comparison because of its central position between T1^21/23DN^ and mature subsets. MZ^CD9+^ displayed the highest number of DE genes, whereas FO-II had the lowest (Figure 1C). There were significant differences in the number of DE genes detected in our data-set compared to ImmGen (Table S1 in Supplementary Material). For example, ImmGen identified seven genes higher in T1 versus T2 and 23 genes higher in T2 versus T1 (FC > 2 cut-off). Our RNA-Seq data identified 375 genes higher in T1^21/23DN^ versus T2^CD21int^ and 326 genes higher in T2^CD21int^ versus T1^21/23DN^ (also FC > 2 cut-off). This clearly demonstrates the utility and advantage of our sorting scheme as well as use of RNA-Seq technology.

Available information suggests that T1 differentiate into T2, which give rise to mature B-cells. To determine B-cell subset relationships, we used the number of DE genes (FC > 2) to construct a distance graph, which suggested three additional differentiation paths (dashed lines): (1) T1^21/23DN^ to MZ^CD9+^, (2) FO-II to FO-I, and (3) FO-II to MZ^CD9+^ (Figure 1D). Scatterplot and PCA analysis illustrates that among our subsets, MZ^CD9+^ was most closely related to FO-II. Surprisingly, T1^21/23DN^ and T2^CD21int^ were almost equally related to MZ^CD9+^ cells (Figures 1E,F). These three potential MZ^CD9+^ precursors shared significant overlap in DE genes relative to MZ^CD9+^ (Figure 1G). This analysis also shows that FO-II are in the middle of a follicular differentiation pathway between T2^CD21int^ and FO-I (Figures 1E,F). Consistently, there was a high degree of overlap in DE genes between T2^CD21int^ and FO-II or FO-I (Figure 1H).

### Distinct transcription programs define transitional and mature B-cell subsets

To systematically identify transcription regulators (TR) (TF plus regulatory genes) that control co-expression of B-cell stage-specific genes, we exploited 1581 TRs recently used to define transcriptional architecture of human hemato- and lympho-poiesis (26). A major shift in the expression of TRs accompanied T1^21/23DN^ to T2^CD21int^ differentiation (Figure 2A). These included Myb, Sp3, and Sox4 at the T1^21/23DN^ stage whereas Myc, Bmyc, Rarα, Nr4a1/Nur77, and Cebpb were prominent at the T2^CD21int^ stage. These TRs are predicted to regulate key biological processes with Myc alone regulating over 19% of DE genes at the T2^CD21int^ stage (Tables S2 and S3 in Supplementary Material).

We also identified TFs relevant for transitional B-cell maturation, e.g., down-regulation of B-cell differentiation TF Gfi1 coincided with differentiation of transitional into mature B-cells (Figure 2D) (27). Conversely, proliferation-promoting Pou6f1 was enriched over three-fold in mature B-cells (Figure 2B) (28). T2^CD21int^ to FO differentiation is regulated mainly by metalion binding TFs (Table S3 in Supplementary Material). Further, more TRs were up-regulated in FO-I relative to FO-II consistent with FO-II being less differentiated (Figure 2G). GeneGo analysis predicted Tbet, Rar, and Titf/Nkx2-1 TFs were required for differentiation into FO-I whereas Nfatc2 is likely to be essential for FO B-cell function (Table S2 in Supplementary Material). Zfp467 and Stat4 were specifically enriched in FO-I and FO-II (Figure 2C) and may therefore selectively regulate FO differentiation and function. Interestingly, Klf2 was predicted to drive FO-II → FO-I terminal differentiation suggesting expression of Klf2 may determine FO-II fate into either FO-I or MZ (Table S3 in Supplementary Material). These data reinforce a T2/FO B-cell differentiation cluster requiring relatively few DE TRs.

T2^CD21int^ to MZ^CD9+^ differentiation required substantial and unique TR rewiring, particularly TFs with zinc-finger and PAS domains (Figure 2E; Table S3 in Supplementary Material). Many DE TRs involved in T1^21/23DN^, T2^CD21int^, or FO-II differentiation into MZ^CD9+^ overlapped suggesting commonality in MZ^CD9+^ fate (Figure 2H). Signature MZ^CD9+^ TRs included those not previously linked to MZ^CD9+^ differentiation and function. One example is Myc binding protein 2 (PAM), which is expressed 4.5-fold higher in MZ^CD9+^ compared to other subsets. We also observed that many TFs [Jund, Junb, Jun, Fos, and Klf2 (29)] were uniquely down-regulated in MZ^CD9+^ (Figure 2D). TFs uniquely up-regulated in MZ^CD9+^ cells were predicted to regulate many genes, including those involved in innate immune response (Table S2 in Supplementary Material). Reduced HDAC and distinct HAT expression profile in MZ^CD9+^ cells was consistent with dramatically altered transcriptome (Figure 2F). Additionally, we observed Nfat TFs (Nfat5, Nfatc1) as well as Nfat activator Nfam1 were particularly enriched in MZ^CD9+^ cells (Table S1 in Supplementary Material). Thus, we have identified previously known and unknown TRs that contribute to MZ^CD9+^ differentiation and function.

### Transcriptome associated with peripheral B-cell tolerance

To systematically define transcriptional programs that regulate alterations in fundamental processes/genes and eventual biological properties of individual B-cell subsets, we identified genes uniquely expressed or repressed termed signature genes (Figure 3; Table S1 in Supplementary Material). T1^21/23DN^ were enriched in mitosis factors including cyclins, microtubule motor activity, nucleoside binding, DNA biosynthesis and recombination, and serine/threonine kinases and were deficient in MHCII, B-cell mediated immunity, positive regulation of NFκB, and cytokine and metabolic activity (Figures 3A,B; Table S3 in Supplementary Material). Given that DNA recombination is critical for receptor editing, we confirmed that T1^21/23DN^ cells uniquely expressed RAG1 and RAG2 (Figures 3C,D). Although previous reports have shown differential RAG expression in splenic transitional B-cells, we observed much greater FC possibly because our sorting scheme excluded CD21^+^ and CD23^+^ cells (30).

Apoptosis and cell cycle control are integral to transitional B-cell tolerance and maturation. Unexpectedly, we did not observe a generalized enrichment of apoptosis and/or cell cycle regulatory genes in any B-cell subset. However, T1^21/23DN^ subset was enriched in select pro-apoptotic genes (Bmf and Apaf1) suggesting their role in negative selection (Figures 4A–C). As expected, apoptosis-resistant T2^CD21int^ cells were enriched for anti-apoptotic genes Bcl-2a1b/d and Bcl-2. Bcl-2 and other anti-apoptotic protein levels largely corresponded with the gene expression (Figure 4D). T1^21/23DN^ cells also expressed higher cell cycle repressors Rb1 and Cdkn1b whereas T2^CD21int^ cells expressed slightly higher Myc-target gene cyclin Ccnd2, a key regulator of proliferation (31) (Figures 4E–H). These findings are consistent with T1^21/23DN^ sensitivity to apoptosis and tolerance checkpoint.

### Transcriptome associated with B-cell maturation and immune competence

Myc is kept in check in T1^21/23DN^ and up-regulated in T2^CD21int^ and beyond (Figure 5A). Due to their significance in stabilizing Myc, we focused on PI3K and Ras signaling (32, 33). We found T1^21/23DN^ cells were significantly deficient for PI3K adaptor protein HCST (DAP10) as well as Ras signaling molecules RasGef1b and RafGef4 (Epac2) (Figures 5C–E). Instead, T1^21/23DN^ cells expressed twofold more PI3K antagonist PTEN (34) and highest catalytic and regulatory subunits of Protein phosphatase 2A (PP2A/Ppp2ca, B56alpha/Ppp2r5a) to increase Myc degradation (Figures 5C,F) (35). Stunted Myc expression in T1^21/23DN^ cells likely prevents autoreactive B-cell survival and maturation. Differentiation past T1^21/23DN^ stage coincided with a significant upregulation of MHCII genes (H2-Ab1/Aa/Eb1/Oa), a hallmark of immune competence (Figure 6A; Table S3 in Supplementary Material). Consistently, T2^CD21int^ were also enriched for Fcer1g, CR2/CD21, complement factors, Icosl and TNFα (Figure 6B; Table S3 in Supplementary Material).

In contrast to T1^21/23DN^, Ppp2ca and Ppp2r5a were lowest in MZ^CD9+^, inversely correlating with the Myc levels (Figure 5F). Enhanced Myc along with a distinct transcriptome, including many signature genes, contribute to the activated state of MZ^CD9+^ cells (Table S1 and S3 in Supplementary Material). Consistent with this state, MZ^CD9+^ were enriched for genes that positively regulate NFκB signaling (Rel, Traf1, Ikβkε, Ikkα, Malt1, PKCβ). Additionally, negative NFκB regulator (IκBβ) as well as proliferation inhibitor Cdkn2d were exclusively down-regulated (31, 36) (Figures 6C,F). However, to prevent overactivity of Myc, MZ^CD9+^ B-cells also have heightened expression of negative regulators Inpp5f, Inpp4a, PTEN, Rap1gap, down-regulated Mapk3, and increased expression of NFκB negative regulators Zfand6, Itch, Rnf11, and most notably Cbl (Figures 5C,E and 6C) (31, 37–39). Overall, these data suggest MZ^CD9+^ cells are primed for quick immune responses including TACI-dependent T-independent immune response (Figure 6E) (40). Finally, MZ^CD9+^ cells were also enriched for MHCI molecules, especially H2-Q6 suggesting MZ^CD9+^ cells are more suited to present certain types of antigens consistent with their known ability to present glycolipid antigens via CD1d (Figure 6A) (13). Interestingly, transcriptome analysis comparing follicular subsets showed FO-I were enriched in histone gene expression while FO-II were enriched for genes involved in lymphocyte and complement activation as well as RNA binding (Figure S1J in Supplementary Material; Table S3 in Supplementary Material). This indicates FO-II share some innate-like qualities with MZ^CD9+^ cells, albeit not as robustly (e.g., CD36 and Perforin-2, Figure 9B), and that they are likely capable of context-dependent plasticity (11).

### CD45-AP regulates MZ B-cell homeostasis

Bioinformatic analysis indicated enhanced regulation of kinase activity in MZ^CD9+^ cells (Table S3 in Supplementary Material). For example, PTPN22 tyrosine phosphatase, a negative regulator of BCR signaling, is expressed highest in MZ^CD9+^ relative to the B-cell subsets analyzed (Figure 7A). Of note, polymorphisms in PTPN22 that render B-cells (and T and innate cells) hyper-responsive and are associated with many autoimmune diseases (41, 42). Conversely, CD45 phosphatase is a major positive regulator of BCR-induced kinase activity (15). Kinase activity is critical for BCR signaling strength, which has been proposed to regulate MZ versus FO-cell fate (11). Our NGS and qRT-PCR data revealed that CD45-associated protein (CD45-AP/Ptprc-ap) was 2.5-fold reduced in MZ^CD9+^ relative to FO-I cells (Figures 7A,B). CD45-AP is one of the most abundantly expressed genes (FO-I FPKM = 1500). Up to 75% of CD45-AP protein complexes with and potentially positively regulates phosphatase activity of CD45, a key positive regulator of BCR signaling (15). However, the role of CD45-AP in B-cell development and homeostasis remains unknown. Therefore, we analyzed splenic B-cell development in mice deficient for CD45-AP (CD45-AP^−/−^) (15). FCM analysis of CD45-AP^−/−^ mice revealed reduced percentage of transitional B-cells (T1 and T2) despite an overall increase in B-cell numbers (Figures 7C–E). Although absolute numbers of FO B-cells were increased, their proportion did not. In contrast, both proportions and absolute numbers of MZ B-cells were significantly increased (Figures 7C–E). Additionally, absence of CD45-AP slightly reduced cell-surface B220 (CD45) potentially further reducing BCR signaling strength (Figure 7F). These results suggest that altered signaling by loss of CD45-AP either facilitates transitional B-cell maturation or survival and/or proliferation of mature B-cell subsets.

### Selective expression and function of IL-9R in MZ B-cells

IL-9R is uniquely enriched in MZ^CD9+^ cells (Figures 8A,B), even when compared to other immune cell populations (43). IL-9R interaction with IL-9 regulates inflammation, humoral immunity, and B1 B-cell expansion (17, 44–48). However, IL-9R function in MZ B-cells remains unknown. Therefore, we tested a role for IL-9R in MZ B-cell development and homeostasis using mice with IL-9R gene deletion and transgenic mice over expressing IL-9. Distribution and numbers of splenic B-cell subsets in IL-9R deficient mice were comparable to controls, precluding a role for IL-9R in splenic B-cell development or homeostasis (Figures 8C,D). Likewise, IL-9 transgenic (Tg) mice did not show any significant alterations in splenic B-cell populations in two different genetic backgrounds, FVB and Balb/c (Figures 8E–G) (17, 48). However, a curious finding was that in IL-9 Tg mice MZ B-cells down-regulated cell-surface expression of CD9 in both genetic backgrounds compared to wild-type control mice (Figures 8H,I). These data are consistent with the highest level of IL-9R expression in MZ B-cells, as predicted by our RNA-Seq and RT-PCR data. Taken together, these results demonstrate that IL-9 and IL-9R are dispensable for B-cell development and homeostasis.

Down-regulation of cell-surface CD9 in MZ B-cells by over-expression of IL-9 suggested that IL-9/IL-9R signaling might be consequential in MZ B-cells. Therefore, we sought to experimentally test whether IL-9R in MZ^CD9+^ B-cells transmits intracellular signals. We measured Stat3 phosphorylation by FCM following IL-9 treatment. Compared to non-stimulated cells, low-dose IL-9 induced robust Stat3 phosphorylation primarily in MZ B-cells (Figures 8J,K). A much smaller increase was also observed in FO B-cells. Activation of pSTAT3 within MZ B-cells was higher in the CD9^+^ than CD9^−^ fraction (Figure 8L). From preferential MZ^CD9+^ B-cell response to IL-9, it seems that this cell population may express highest cell-surface IL-9R protein, consistent with highest IL-9R transcript levels (NGS). Additional experiments showed that in addition to Stat3, IL-9 induced phosphorylation of Stat1 and Stat5 in MZ B-cells. Since, these experiments were carried out with B-cells isolated from Balb/c mice, the results also demonstrate that selective responsiveness of MZ B-cells to IL-9 is independent of the genetic background (Figures 8M–O). To our knowledge this is the first report to demonstrate IL-9/IL-9R signaling in MZ B-cells. However, additional experiments are needed to determine the effects of this signaling on MZ B-cell function.

### MZ^CD9+^ cells selectively express genes associated with innate immunity

Although MZ B-cells have been proposed to have innate-like properties, a comprehensive transcriptome analysis of these characteristics has not been performed (13, 49). Our analysis of innate immune sensors revealed that MZ^CD9+^ cells are enriched in the RNA sensing molecules Tlr3, Tlr7, and Nlrc3/Nod3, the bacterial peptidoglycan sensors Nod1/2/3 and Nlrc4 and the DNA sensors Tlr9 and Prkdc (Figures 9A–E) (50). Innate effector molecules such as respiratory burst oxidase gene Cybb, autophagy associated gene Pik3r4/p150, and the recently identified intracellular bactericidal Perforin-2 are also highly enriched in MZ^CD9+^ cells (Figure 5C and Figures 9B,F) (51, 52). MZ^CD9+^ cells are also enriched for IKKε, which provides a critical link to innate immune pathways under NFκB control. To our knowledge, this is the first report to suggest a biased use of this innate pathway in MZ^CD9+^ B-cells (Figures 6C,F) (53).

We chose TLR3 for functional assessment because it is exclusively expressed in MZ^CD9+^ cells. We show that in total B-cells TLR3 agonist Poly(I:C) was unique among endosomal TLR agonists by inducing a threefold increase in Perforin-2/MPEG1 transcript (Figure 9G). Using sorted cells we showed that MZ^CD9+^, but not FO-I cells, exclusively induced Perforin-2 (Figure 9H). These results show that MZ^CD9+^ cells uniquely and functionally express TLR3, which can endow bactericidal function to MZ^CD9+^ B-cells through Perforin-2 induction.

## Discussion

This study is the first to report the mRNA transcriptome obtained by RNA-Seq of mouse splenic B-cell subsets (43, 54–58). Our data support the hypothesis that MZ^CD9+^ subset may arise from multiple precursors. Differentiation from T1^21/23DN^ would theoretically bypass CD23 expression (59). Further support for T1^21/23DN^ to MZ^CD9+^ pathway comes from shared deficiency of N-regions and an increase in TLR9 and Aiolos expression, both of which promote MZ differentiation (60–62). While FO-II could differentiate into MZ, they are most closely related to FO-I. PCA analysis additionally supported FO-II to be an intermediate of FO-I subset, possibly mediated by Klf2.

Enhanced expression of pro-apoptotic gene (Bmf) in T1^21/23DN^ relative to T2^CD21int^ supports the notion that autoreactive B-cell are deleted by mitochondrial pathway of apoptosis at the T1-checkpoint (63). Our novel finding that Serine incorporator (Serinc5) is exclusively expressed in T1^21/23DN^ cells may provide an additional mechanism of cell death by altering membrane composition and BCR signaling (64) (Figure 3A). Furthermore, we found expression of genes associated with DNA recombination (RAG1, RAG2, Ligase 4) exclusively in T1^21/23DN^ cells suggesting receptor revision. These data suggest apoptosis and receptor revision contribute to peripheral tolerance at the T1-checkpoint.

We also found that up-regulated T1^21/23DN^ signature gene Akap12 may uphold the T1^21/23DN^ stage by interfering with activation, migration, and proliferation (Figure 3A) (65). Consistently, it is dramatically down-regulated in proliferative T2^CD21int^ cells. To our knowledge, this is the first report to identify down-regulation of Akap12 as an indicator of proliferation in splenic B-cells.

Myb to Myc switch from T1^21/23DN^ to T2^CD21int^ was highly consequential as these TFs regulated a substantial proportion of DE genes. We predict Rarα expression contributes to Myb down-regulation (66). Onset of Myc expression in T2^CD21int^ cells profoundly influenced gene expression comprising one-fifth of all DE genes (67) conferring metabolic fitness and likely driving survival, proliferation and differentiation of T2^CD21int^ into mature B-cells (4, 31, 68, 69). Consistently, our data show that MZ^CD9+^ expressed highest levels of Myc as well as Myc binding/regulatory proteins PAM and Max (Figure 5A). Consistent with their resting state, FO-I cells express the least Myc of mature subsets, but express highest Bmyc, which we suggest transcriptionally represses Myc (70). Both heightened expression of Bmyc in FO-I and its potential function in B-cells have not been previously reported.

Our data also suggest uncoupling of the BCR from growth, metabolic, and Myc pathways (PI3K and Ras/ERK) contribute to the distinct T1^21/23DN^ cell biology. This may be explained by our novel finding that the PI3K pathway may be may not be fully activated in T1^21/23DN^ cells due to severely reduced levels of the Dap10 adapter for PI3K (71). This genetic data are supported by experimental evidence showing T1 cells only weakly activate the PI3K pathway (via Akt phosphorylation) (3, 5). Additionally, increased expression of negative regulators of BCR signaling PTEN, CD72, and PP2A would further limit the PI3K/Myc pathway in T1^21/23DN^ cells (34, 35, 72). Differentiation into T2^CD21int^ is also accompanied by Bcl-2 transcript and protein. Heightened expression of both Myc and Bcl-2 is a highly potent combination (highest in MZ^CD9+^) promoting survival during Myc-driven proliferation and frequently occurs in various B-cell lymphomas (73, 74). We suggest that survival at the T2^CD21int^ stage is also supported by TNFα as T2^CD21int^ and mature B-cells express only pro-survival TNFR2 (75). Together, these data point to a previously unknown PI3K- and Myc-driven transcription program, facilitated by TNFα, which distinctly controls metabolic activity in the two transitional B-cell subsets to allow negative selection in T1^21/23DN^ and proliferation and differentiation in T2^CD21int^ B-cells.

With the gain of survival and proliferation potential, T2^CD21int^ cells begin to express MHCII and its transcriptional activator, CIITA as well as the immune modulator Icosl. While the altered gene expression endows T2^CD21int^ cells immune competence, it also prevents inadvertent B-cell mediated T cell activation, which can lead to autoimmune diseases (76). As T2^CD21int^ cells are poised for further differentiation in the splenic follicles, they express LTα and LTβ and facilitate follicular architecture and secondary lymphoid organogenesis (Table S1 in Supplementary Material) (77, 78).

Our analysis of CD45-AP^−/−^ mice revealed an increase in the percentage and numbers of total B-cells due to an increase in mature B-cells, particularly MZ type, similar to CD45^−/−^ mice (79, 80). However, in contrast to CD45, CD45-AP appears to restrict transitional B-cell differentiation, especially to MZ B-cell fate or it is required for maintaining homeostasis within the mature B-cell compartment. Additionally, reduced B220/CD45 surface expression in CD45-AP^−/−^ B-cells suggested that CD45 and CD45-AP are reciprocally dependent for protein stability (81–85). A reduction in CD45 and consequent phosphatase activity would reduce BCR signal strength. Despite conflicting reports concerning CD45-AP’s role in receptor signaling (15, 81, 83, 86–89), we propose CD45-AP effects BCR signaling either in transitional cells to prevent their premature entry to the mature B-cell pool or their survival and proliferation. Thus, CD45-AP regulates splenic B-cell homeostasis (79).

Our transcriptome data showing significantly higher IL-9R together with high levels of heterodimer partner IL-2Rγ suggested a potential function for IL-9R in MZ^CD9+^ B-cells. While IL-9R did not play a role in MZ B-cell development, it may play a role in MZ B-cell function as exposure to IL-9 induced phosphorylation of Stat proteins. Activation of Stat proteins, particularly Stat3, is important in host defense (90). Consistently, over-expression of IL-9 has previously been shown to result in increased immunoglobulins before and after immunization (17, 44–46). These findings suggest a potential role for IL-9R-dependent activation of MZ B-cells in immune response (91). Although MZ^CD9+^ B-cells specialize in TI antibody responses, elevated IL-9R suggests that the TD antibody responses may also be differently regulated in this B-cell subset. Consistently, key regulators of antibody response in FO B-cells (IL-4R and IL-21R) are specifically down-regulated in MZ B-cells (Table S1 in Supplementary Material).

A striking finding was that long-term activation of MZ B-cells by IL-9 in transgenic mice dramatically down-regulated CD9 surface levels possibly relating to its function in BCR signaling, migration, adhesion and homing (92–94). However, CD9 deficiency does not alter B-cell development or humoral immunity perhaps due to functional redundancy (95). Taken together, IL-9/IL-9R induction of Stat pathway and down-regulation of CD9 suggests that IL-9R contributes to MZ B-cell function.

Accumulating evidence indicates MZ B-cells function in both innate and adaptive immunity (13) and we identified many DE transcripts with unknown functions in this subset. One such molecule was PAM, which has not been previously linked with MZ B-cell function. This E3 ubiquitin ligase mediates mTOR activation through sphingosine-1-phosphate receptors, of which S1p1 and S1p3 are both highly enriched in MZ^CD9+^ cells (Figure 5B) (96, 97). Given that mTOR promotes B-cell activation, maturation, antibody production, and survival, we speculate PAM bridges mTOR activation with sphingosine receptor signaling imparting MZ^CD9+^ with unique functionality (98). We provided evidence that MZ^CD9+^ cells possess unique innate sensing ability through expression of various PAMP receptors such as bactericidal Perforin-2. Further, we showed that TLR3 induction exclusively induced Perforin-2 in MZ^CD9+^ cells (51, 52). Thus, through a unique transcription program, MZ^CD9+^ cells become hardwired to recognize infectious agents and respond quickly to bridge the adaptive immune response.

In summary, our data have identified several genes and gene clusters, which have not previously been linked to specific splenic B-cell subsets. These data revealed potential novel developmental relationships among splenic B-cell populations, and indicated receptor revision in T1^21/23DN^ may contribute to peripheral tolerance. The first major shift in the transcription program accompanied T1^21/23DN^ → T2^CD21int^ differentiation, which was dominated by Myc and PI3K/Ras pathways indicating enhanced metabolic activity, survival (via Bcl-2) and proliferation and these alterations were largely shared with mature B-cell subsets. Our analysis also demonstrated that CD45-AP is important for peripheral B-cell homeostasis while IL-9R participates in MZ^CD9+^ cell function. Highly selective expression and function of IL-9R suggests that in MZ^CD9+^ B-cells, TD antibody responses are regulated via mechanisms distinct from FO B-cells. Further, MZ^CD9+^ cells expressed genes that are known to confer innate effector functions as exemplified by expression of PAMPs, TLR3, and Perforin-2. Thus, MZ^CD9+^ B-cells are uniquely suited for TI antibody response, may distinctly regulate TD antibody response and possess broader innate immune capabilities than previously appreciated.

## Author Contributions

EK, KH, AT, JR, and WK designed research; EK, MH, KH, JL, IC, JW, and DD performed research; EK, DS, ESC, EC, and WK analyzed data; KH edited text; EK and WK wrote the paper.

## Conflict of Interest Statement

The authors declare that the research was conducted in the absence of any commercial or financial relationships that could be construed as a potential conflict of interest.

## Supplementary Material

The Supplementary Material for this article can be found online at http://www.frontiersin.org/Journal/10.3389/fimmu.2015.00030/abstract

## Footnotes

^1^http://www.bioinformatics.babraham.ac.uk/projects/fastqc/

^2^http://www.bioconductor.org/

^3^http://www.r-project.org/
