# Corrigendum: Distinct Transcriptomic Features Are Associated with Transitional and Mature B-Cell Populations in the Mouse Spleen

**DOI:** 10.3389/fimmu.2016.00267

**Published:** 2016-07-04

**Authors:** Eden Kleiman, Daria Salyakina, Magali De Heusch, Kristen L. Hoek, Joan M. Llanes, Iris Castro, Jacqueline A. Wright, Emily S. Clark, Derek M. Dykxhoorn, Enrico Capobianco, Akiko Takeda, Ryan M. McCormack, Eckhard R. Podack, Jean-Christophe Renauld, Wasif N. Khan

**Affiliations:** ^1^Department of Microbiology and Immunology, Miller School of Medicine, University of Miami, Miami, FL, USA; ^2^Center for Computational Science, University of Miami, Miami, FL, USA; ^3^Ludwig Institute for Cancer Research, Brussels Branch, Brussels, Belgium; ^4^de Duve Institute, Université Catholique de Louvain, Brussels, Belgium; ^5^Department of Pathology, Microbiology and Immunology, Vanderbilt University School of Medicine, Nashville, TN, USA; ^6^Hussman Institute for Human Genomics, University of Miami, Miami, FL, USA; ^7^Department of Pathology and Immunology, Washington University School of Medicine in St. Louis, St. Louis, MO, USA

The authors regret that Ryan M. McCormack and Eckhard R. Podack shown above were inadvertently omitted from the author list.

The correct author list is shown below including the authorship statement.

Eden Kleiman, Daria Salyakina, Magali De Heusch, Kristen L. Hoek, Joan M. Llanes, Iris Castro, Jacqueline A. Wright, Emily S. Clark, Derek M. Dykxhoorn, Enrico Capobianco, Akiko Takeda, Ryan M. McCormack, Eckhard R. Podack, Jean-Christophe Renauld, and Wasif N. Khan

The original article has been updated.

## Author Contributions

Eden Kleiman, Daria Salyakina, Kristen L. Hoek, and Wasif N. Khan designed research, performed experiments, analyzed data, and wrote the manuscript; Magali De Heusch, Joan M. Llanes, Iris Castro, Jacqueline A. Wright, Emily S. Clark, Derek M. Dykxhoorn, and Ryan M. McCormack performed and analyzed the experiments; Akiko Takeda, Jean-Christophe Renauld, and Enrico Capobianco provided the materials and discussed results, and Eckhard R. Podack discovered and named Perforin-2, and interpreted and discussed the results.

The authors apologize for this mistake. The correction does not affect the scientific validity of the results.

## Conflict of Interest Statement

The authors declare that the research was conducted in the absence of any commercial or financial relationships that could be construed as a potential conflict of interest.

