# Supplementary figures and images for: Distinct Transcriptomic Features are Associated with Transitional and Mature B-Cell Populations in the Mouse Spleen
Source: Front Immunol. 2015 Feb 11;6:30. doi: 10.3389/fimmu.2015.00030 (PMC4324157; doi:10.3389/fimmu.2015.00030)

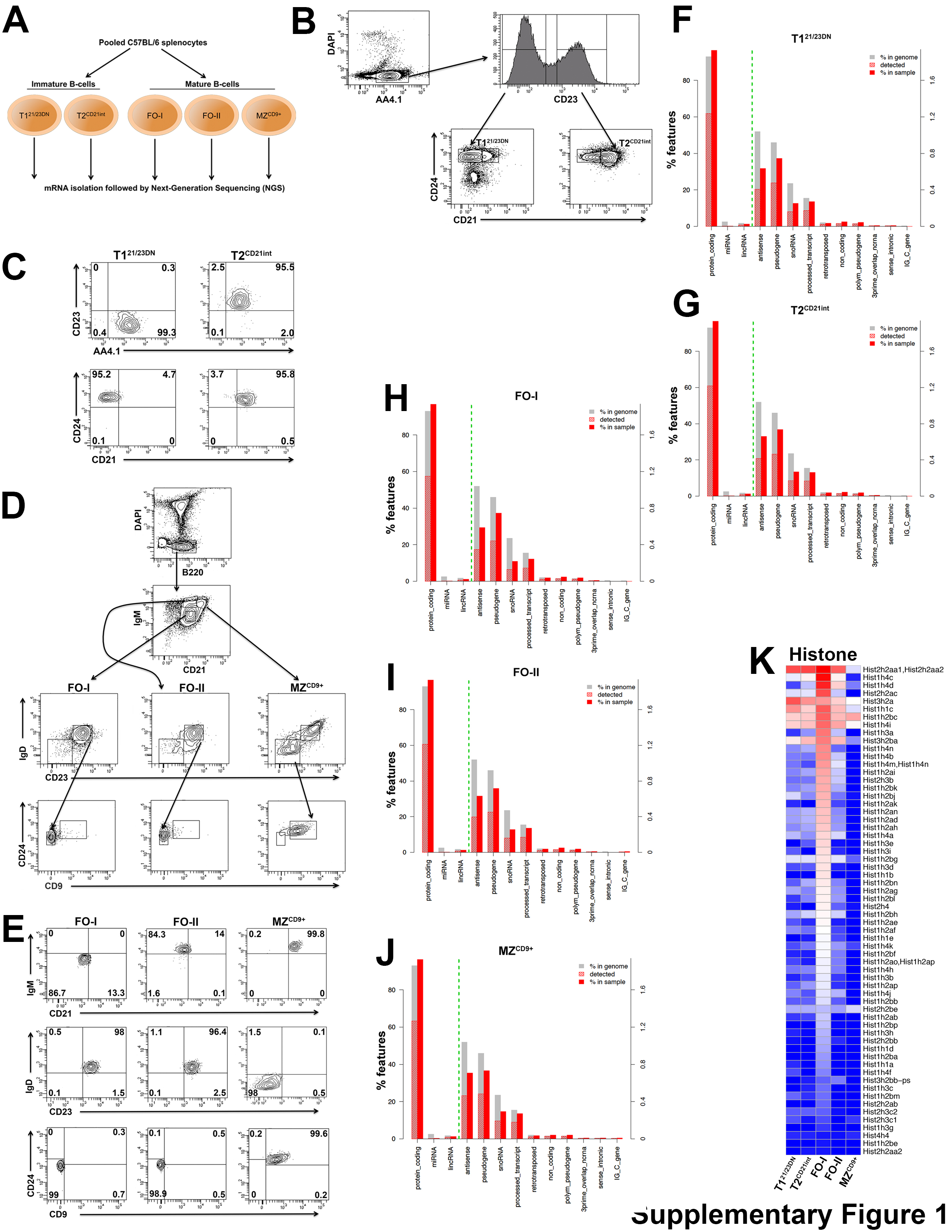

Supplement: Supplementary file 4 [file Image_1.JPEG]
